# RNAi-Mediated Resistance Against Viruses in Perennial Fruit Plants

**DOI:** 10.3390/plants8100359

**Published:** 2019-09-22

**Authors:** Khushwant Singh, Chris Dardick, Jiban Kumar Kundu

**Affiliations:** 1 Division of Crop Protection and Plant Health, Crop Research Institute, Prague 161 06, Czech Republic; singh@vurv.cz; 2 United States Department of Agriculture, Agricultural Research Service, Appalachian Fruit Research Station, Kearneysville, WV 25430, USA; Chris.Dardick@ars.usda.gov

**Keywords:** sRNA, viruses, resistance, next generation sequencing, perennial plants

## Abstract

Small RNAs (sRNAs) are 20–30-nucleotide-long, regulatory, noncoding RNAs that induce silencing of target genes at the transcriptional and posttranscriptional levels. They are key components for cellular functions during plant development, hormone signaling, and stress responses. Generated from the cleavage of double-stranded RNAs (dsRNAs) or RNAs with hairpin structures by Dicer-like proteins (DCLs), they are loaded onto Argonaute (AGO) protein complexes to induce gene silencing of their complementary targets by promoting messenger RNA (mRNA) cleavage or degradation, translation inhibition, DNA methylation, and/or histone modifications. This mechanism of regulating RNA activity, collectively referred to as RNA interference (RNAi), which is an evolutionarily conserved process in eukaryotes. Plant RNAi pathways play a fundamental role in plant immunity against viruses and have been exploited via genetic engineering to control disease. Plant viruses of RNA origin that contain double-stranded RNA are targeted by the RNA-silencing machinery to produce virus-derived small RNAs (vsRNAs). Some vsRNAs serve as an effector to repress host immunity by capturing host RNAi pathways. High-throughput sequencing (HTS) strategies have been used to identify endogenous sRNA profiles, the “sRNAome”, and analyze expression in various perennial plants. Therefore, the review examines the current knowledge of sRNAs in perennial plants and fruits, describes the development and implementation of RNA interference (RNAi) in providing resistance against economically important viruses, and explores sRNA targets that are important in regulating a variety of biological processes.

## 1. Introduction

Small RNAs (sRNAs) are single-stranded, noncoding RNA molecules 20–30 nucleotides (nt) long. They are conserved in most eukaryotes and regulate gene expression in a sequence-specific manner either transcriptionally or post-transcriptionally [1,2]. Classified by differences in their mechanisms of production, functions, and features, sRNAs include microRNAs (miRNAs), small interfering RNA (siRNA), Piwi-interacting RNA (piRNA), repeat-associated siRNAs (ra-siRNAs), small nucleolar RNA (snoRNAs), phased siRNAs (pha-siRNAs), *cis* and *trans* natural antisense transcript siRNAs (*cis*- and *trans*-nat siRNAs), tRNA-derived small RNA (tsRNA) and small rDNA-derived RNA (srRNA) [3].

Plant viruses pose major threats to a broad range of crops, causing economic losses (10–15%) that rank second to those caused by other pathogens [4]. Plant viruses enter host plants through openings, wounds or by the feeding action of insect vectors, then replicate in host cells, move from cell to cell, and spread long distances via the phloem [5]. Due to complex epidemiological factors associated with virus disease outbreaks, such as evolution of virus at high pace, vector migration dynamics, and unpredictable virus host-range expansions, it is difficult to develop an efficient disease management strategies [6]. The deployment of genotypes with virus resistance has proven to be the most effective strategy [7,8,9].

**Figure 1:**
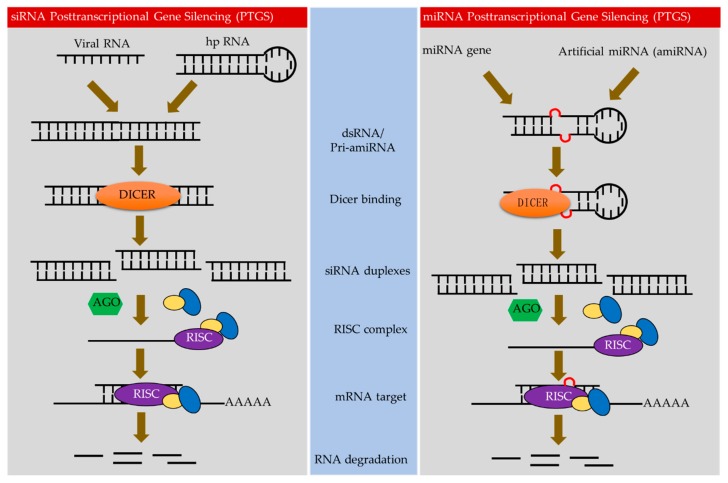
Types of RNA-mediated gene silencing in perennial plants. In perennials plants, antiviral silencing has been accomplished through sense-gene-induced posttranscriptional gene silencing (S-PTGS), artificial miRNA-induced PTGS (AMIR-PTGS) and hairpin-RNA-induced PTGS (hp-PTGS).

During host–virus interactions, viruses must create a suitable environment to replicate, which involves manipulating the host cellular machinery and ultimately transforming the host cells into “viral factories” [10]. Viral-encoded proteins interact with host transcription machinery, DNA replication and proteins related to cell division, defense, cell redox homeostasis and plant metabolic processes [11]. These host–virus interactions also trigger antiviral responses from the host. Plants use several mechanisms to challenge virus infection, including RNAi, systemic acquired resistance, hypersensitive response (HR), and DNA methylation. Resistance can also be achieved when key host proteins are absent or have structural changes that prevent association with viral proteins [12]. sRNA-based strategies to engineer resistance against viruses, including hairpin RNA-mediated interference-based strategies (hpRNAi), artificial microRNA (amiRNAs), and artificial *trans*-acting siRNAs (atasiRNA) [8] are highly effective and have been used to develop virus-resistant crops [13,14,15,16] (Figure 1).

## 2. The Molecular Mechanism Underlying RNA Silencing in Plants

RNA silencing refers to sequence-specific gene-silencing mechanisms, which is entangled in the development and maintenance of genome integrity and antiviral defense [17]. RNAi technology has been implemented to decipher gene function, as well as to generate plants with improved or novel traits by maneuvering desirable or undesirable genes.

The class of sRNAs that play a key role in directing RNAi processing are the short interfering RNA molecules (siRNAs; also called small interfering RNAs). SiRNAs comprise of 21–26 bp and produced by the cleavage of longer double-stranded RNAs (dsRNAs) via Dicer or Dicer-like proteins (DCL) (Figure 1) [18]. Four Dicer-like nucleases (DCLs) with distinct, hierarchical, and overlapping functions in sRNA biogenesis have been reported in angiosperms [19]. Mature sRNAs associate with Argonaute (AGO) to form the core of the RNA-inducing silencing complex (RISC). sRNAs guide the RISC complex to complementary target RNA molecules [20]. Finally, the silencing complex downregulates complementary RNA targets by either cleaving target mRNAs or repressing translation [21,22]. In addition, some sRNAs can induce methylation or histone modification of target genomic loci [23].

Plant sRNAs that mediate gene silencing have been generally categorized as either microRNAs (miRNAs) or siRNAs. The miRNAs are encoded by genes (referred to as MIRs) that produce hairpin-like RNA structures that are cleaved by DCLs, while siRNAs are produced by DCLs from host RDR-dependent RNA polymerase (RDR) [24]. Both miRNAs and siRNAs are assorted by AGOs, mostly based on the size of sRNA as well as identity at 5′-nucleotide (nt), to form RISC complex that mediate post-transcriptional gene silencing (PTGS) via sRNA-directed mRNA cleavage or translational repression and TGS via sRNA-directed DNA methylation [25]. Because sRNAs play an essential role in targeting RISC complexes to complementary sequences, their composition and abundance is critical for the efficacy of silencing individual RNA targets.

## 3. sRNA-Mediated Resistance in Perennial Fruits Against Viruses

Biotechnology exploiting sRNA for crop resistance against viruses [15,16,26] has been developed for several perennial plants, and a few crops that are complete resistance to virus have been regulated for commercial purpose [21,27,28,29]. In perennials, antiviral silencing has been achieved through sense-gene-induced posttranscriptional gene silencing (S-PTGS), artificial miRNA-induced PTGS (AMIR-PTGS) and hairpin-RNA-induced PTGS (hp-PTGS) [27] (Figure 1).

### 3.1. Sense Gene-Induced PTGS

S-PTGS, has been used to modify plant traits of economic importance and analyze gene function [30]. In S-PTGS, transcripts from transgene loci recruit RNA-dependent RNA polymerase 6 (RDR6) to synthesis complementary RNA strands, which leads to the processing of siRNAs from dsRNAs by DCLs. Single-stranded siRNAs are then integrated into AGO1, that mediates degradation of the target mRNA in the plant cytoplasm [31] (Figure 1).

S-PTGS resistance depends on sequence homology between corresponding viral genome and a transgene. Transgenic papaya (*Carica papaya* L.) line 55-1 was created to express the coat protein (CP) gene of the mild strain of the papaya ringspot virus (PRSV) isolates from Hawaii [32,33]. Transgenic papaya cultivars SunUp and Rainbow were produced. SunUp is homozygous while Rainbow is hemizygous for the CP gene. PRSV transgenic SunUp and Rainbow are largely influenced by CP transgene dosage, sequence homology between the transgene and the isolates and various stages of plant development [34]. However, transgenic papaya cultivars from different geographical regions have distinct levels of resistance against PRSV. For instance, isolates from the Florida, Bahamas and Mexico showed delayed and mild symptoms, as compared to the isolates from Brazil and Thailand that showed delayed symptoms, however PRSV ultimately surpasses their resistance [26].

**Figure 2:**
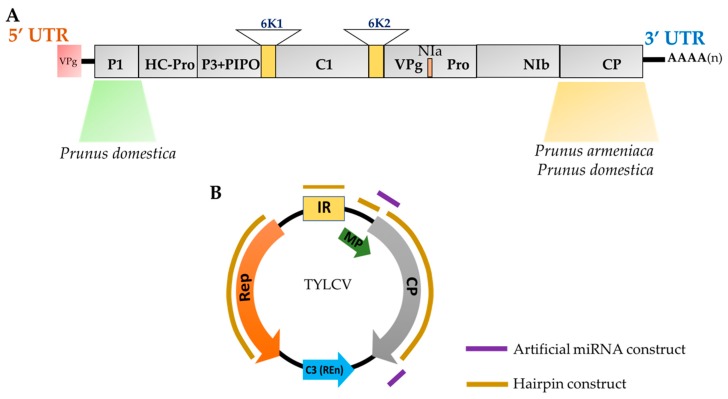
Viral targets for sRNA-mediated resistance against viruses (SMR). A) genomic map including UTR’s of the *Plum pox virus* (PPV). Schematic representation of PPV sequences employed for obtaining PPV resistant plants. The viral P1 protein and CP gene sequences were used to obtain resistance against *Prunus domestica* [40,41,42,43,44,45,46,47], while CP sequence construct was used to transform *Prunus armeniaca* [48] B) represents the genomic map of *Tomato yellow leaf curl virus* (TYLCV) and *Tomato yellow leaf curl virus*-Oman (TYLCV-OM). Artificial miRNA construct [49] and hairpin constructs [50] from non-coding intergenic region (IR), coat protein (CP), movement protein (MP) and replication-associated protein (Rep) were utilized for obtaining resistant plants.

Transgenic plum clone, C5/HoneySweet was transformed with the sense *Plum pox virus*-D (PPV-D) coat protein (CP) transgene gene and is highly resistant to *Plum pox virus* (PPV) [35] (Figure 2). Transgenic HoneySweet carries multiple and rearranged viral CP copies, expresses significantly low level of CP mRNA and does not accumulate detectable amounts of the CP [36]. The HoneySweet CP transgene is methylated, and the siRNAs generated are specific to the CP transgene [35]. The siRNA duplex (21–26 nt) was detected in healthy and PPV-inoculated virus-free HoneySweet plants [37]. Symptomatic HoneySweet has a higher level of 21- and 22-nt siRNAs than 25–26-nt siRNAs, which suggests that PTGS in HoneySweet plants was suppressed [38]. However, we do not yet know how these siRNAs influence the RNAi silencing mechanism in HoneySweet or what level of abundance of siRNAs is specific to the CP transgene. High-throughput sequencing of small RNAs (HTS-sRNA) is underway to decipher the mechanism of RNAi-mediated virus resistance in Honeysweet (Callahan A, unpublished data, USDA, Agricultural Research Service, Appalachian Fruit Research Station, Kearneysville, West Virginia).

In tomato, incorporation of a partial polygalacturonase (PG) transgene causes S-PTGS during the ripening of tomato fruits, leading to the accumulation of PG siRNA [39]. When this silencing-induced PG transgene locus was inserted into a tomato variety with a lower level of endogenous PG gene expression during ripening, the endogenous PG gene was strongly suppressed, but the PG transgene was not. This result demonstrates that S-PTGS is linked to the higher copy number of the transgene and/or particular transgene locus as compared to the endogenous gene.

### 3.2. Intron-Spliced Hairpin RNA-Induced PTGS (ihpPTGS)

To confer resistance against PPV in stone fruits, many constructs expressing PPV-derived ihpRNAs were generated that can efficiently induce PTGS. The first PPV ihpRNA construct was developed against the *VPg-P1* gene by Pandolfini et al. [51]. In stone fruits, most of the tested ihpRNA constructs originated from the viral P1 protein and CP genes [44,45,46]. In *Nicotiana benthamiana*, ihpRNAs constructs from P1 and helper component protease (HC-Pro) were found to confer resistance against PPV [52]. ihpRNAs were effective against various PPV isolates i.e PPV-D and PPV-M [15]. More recently, ihp-RNA approach was evaluated to manipulate the expression of the recessive viral resistance gene required for viral translation, eukaryotic initiation factor 4E (*eIF4E*) or its isoform *eIF(iso)4E* from *Prunus domestica* [53]. These results not only showed the involvement of *eIF(iso)4E* in PPV infection in plums but also silencing of *eIF(iso)4E* can lead to PPV resistance in *Prunus* species. Similarly, a hairpin sequence designed from the coat protein fragments of the *Prunus necrotic ringspot virus* (PNRSV: PNRSV-hpRNA) was highly efficient in defending transgenic cherry rootstocks from PNRSV damage [54]. The hpRNA in the construct (*pART27-PNRSV-hpRN*) was part of the RNA3 sequence of PNRSV genome. A hpRNAi construct to express dsRNA homologous to sequences of the coat protein gene, intergenic region (IR), replication-associated gene, and V2 gene of *Tomato yellow leaf curl virus-Oman* (TYLCV-OM) was developed to deliver resistance combating geminiviruses in tomato [50] (Figure 2). The resistance in this instance was not immunity, but disease severity and virus titer were reduced. In contrast, transgenic banana (*Musa* spp.) expressing ihpRNA transcripts of the viral replication initiation protein (Rep protein) of *Banana bunchy top virus* (BBTV) gave complete resistance to the virus [55].

### 3.3. Artificial miRNA-Induced PTGS (AMIR)

Artificial miRNAs (amiRNAs) can also be used to guide the silencing of target genes with high specificity and have been adapted to produce virus-resistance plants [12] (Figure 1). The amiRNA strategy for obtaining virus-resistant transgenic plants has worked against various viruses including Cucumber mosaic virus (CMV), Potato virus Y (PVY), Potato virus X (PVX), watermelon silver mottle virus (WSMoV), tomato leaf curl virus New Delhi virus (ToLCNDV), cotton leaf curl Burewala virus (CLCBV) and wheat streak mosaic virus (WSMV) [12,56,57]. Vu et al. [49] designed amiR-AV1-3, which targeted the mid region of the AV1 (coat protein) transcript, and amiR-AV1-1, which targeted the overlapping region of the AV1 and AV2 (pre-coat protein) transcripts, of ToLCNDV. According to their study, the T2 generation plants of the transgenic tomato that expressed amiRAV1-1 were tolerant to ToLCNDV, whereas those expressing amiR-AV1-3 were only moderately tolerant.

## 4. Graft Transmissibility of sRNA Resistance in Fruit Trees

Using transgenic rootstock to express sRNAs to trigger RNA silencing in nontransgenic scions is an effective way to tackle challenges in developing viral resistance in fruit rootstocks. To date, few studies have investigated for the virus resistance in perennial woody plants that involved the production and transportation of hpRNA-derived siRNAs [58]. Transgene-derived siRNAs from a hairpin sequence of the partial coat protein of PNRSV were noticeably effective against PNRSV in protecting transgenic cherry rootstocks [54], and transgene-derived siRNAs induced systemic silencing in nontransgenic scions in grafted cherry trees [58]. siRNAs profiles generated using HTS-sRNA from transgenic cherry rootstocks and PNRSV-inoculated transgenic cherry rootstocks suggested that hpRNA accumulated 24-nt siRNAs in the transgenic rootstock. Global analysis of the fruit transcriptome in white and red genotypes of strawberry showed the downregulation of 33 genes. Transcript levels of strawberry endogenous genes *Fragaria ananassa* chalcone synthase (FaCHS) and *F. ananassa O*-methyltransferase (FaOMT) were equally reduced [59].

In apple, with transgenic rootstock overexpressing hrp-*gusA* gene construct and scion T355, *gusA* was expressed in T355 scions *in vitro*, but not in T355 scions grown in the greenhouse [60]. Nature of the targeted gene and the sRNAs produced by the transgene greatly influence graft transmission of viruses which might result in weak infection with lower efficiency. For example, systemic silencing was only achieved in leaves of tobacco when the glutamate-1-semialdehyde aminotransferase gene (*GSA*) was targeted for graft-transmissible siRNA silencing, suggesting weak transmission of *Commelina* yellow mottle virus (CoYMV) [61]. Ali et al. [62] showed RNA silencing of *NtTOM1* and *NtTOM3*, endogenous genes in tobacco that are essential for tobamovirus multiplication, allowed high resistance against several tobamoviruses because very low levels of viruses were detected in both the rootstocks and scions. However, potential steps towards enhancing the efficiency of silencing in grafted trees include (I) characterization of gene targets that enable efficient silencing through grafts in fruit trees, (II) using transgenic stock that is competent for transporting a specific siRNA, which might ensure a novel approach for improving the agricultural characteristics of a grafted scion cultivar and (III) generating sRNA in the rootstock to achieve stronger silencing [63].

## 5. Viral Targets for sRNA-Mediated Resistance Against Viruses (SMR)

The viral targets for SMR incorporate virus and the gene/s that are directed by sRNAs produced by the transgene. Tospoviruses (genus *Orthotospovirus*), *Potyvirus*, *Closterovirus*, and *Geminiviruses* (genus *Begomovirus*) are among the most studied virus genera exploited for SMR. Specific coding regions of these viral genomes including the CP, movement protein (MP), nuclear protein, RNA-dependent RNA polymerase (RdRP), viral suppressor of RNA silencing (VSR) protein and replication-associated proteins (RAP) are the most common and effective targets for SMR [10,39,64] (Figure 2). In addition to the coding region, 5′ and 3′ untranslated regions (UTRs) of viral genomes can also yield efficient antiviral silencing [41,44,45,47]. The *Cauliflower mosaic virus* promoter (CaMV35S) has been used extensively to investigate the silencing transcript expression to generate siRNAs or miRNAs that target viruses [65].

## 6. Identification of sRNAs in Perennial Species Using Next-Generation Sequencing (NGS)

**Figure 3:**
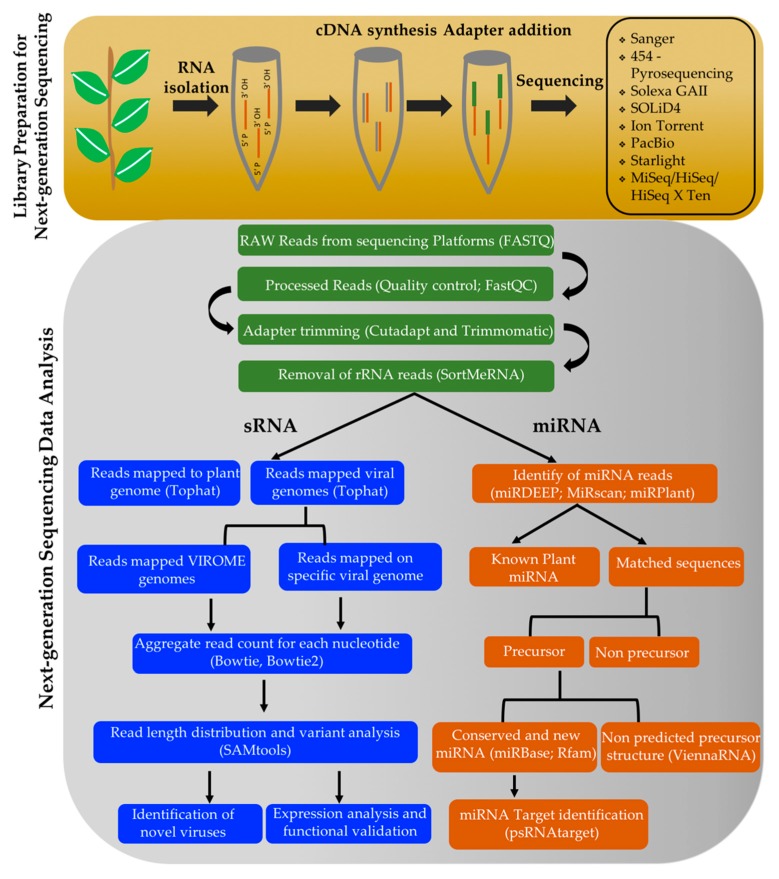
Computational approaches to identify sRNAs in perennial plants using high-throughput sequencing technology.

Collectively, numerous studies have shown various degrees of success in engineering virus-resistant plants using sRNA-producing transgene cassettes. Improving these technologies will require a greater understanding of how various sRNA strategies differ in their efficacy. Such differences likely are driven mainly by the abundance, size, and distribution of sRNAs and their targets and the overall context of the naturally occurring host and viral-derived sRNA pools. Such information can be obtained using HTS-sRNA strategies. Large numbers of native sRNAs have been identified in perennial plants by sequencing small RNA libraries ([65,66]; A. Callahan, unpublished data, USDA, Agricultural Research Service, Appalachian Fruit Research Station, Kearneysville, West Virginia). Various computational approaches have been exploited to identify sRNAs, and they have been functionally validated in few perennial species (Figure 3).

### 6.1. Prunus

Zhang et al. [66] identified 22 novel miRNAs in peach using NGS. Eight candidate miRNAs were validated experimentally using UTRs miRNA-RACE PCR reactions and sequence-targeted cloning in peach leaves, flowers, and fruits at various stages of development. Expression of the novel miRNAs is tissue-specific, and two precursors, *ppe-miR171a* and *ppe-miR171b*, of the miR171 family were found. In another study, peach sRNAs (miRNAs) from different tissues were comprehensively analyzed and characterized for their expression in roots, leaves, flowers and fruit using sRNA-seq and RNA blots [67]. Size distribution analysis showed that 90% of sRNAs belong to 20 to 24 bp, with the 24-nt class most abundant. Furthermore, peach sRNA sequences were classified into two major families: conserved miRNAs (23 miRNA families) and less-conserved miRNAs (24 miRNA families) [68]. Most of the conserved miRNAs, such as *miR156-miR169*, *miR319*, *miR390* and *miR396*, are expressed at high levels in all tissue i.e., leaf, flower, fruit, root, and bark tissues of peach as compared less-conserved miRNAs such as *miR828*, *miR858* and *miR2118* [67].

In total, 47 novel peach-specific miRNAs were identified from 134 loci and expressed at low levels, which varied in different tissues. Most of the peach-specific miRNA (below 40) found to be originated from a single locus, and the rest matched 2 to 15 loci. Overall, more than 65% miRNAs of peach mapped to the sense strand while ~35% mapped to the antisense strand. The miRNA precursors ranges from 90 to 130 bp. Some miRNAs (miRC26) were observed to be highly accumulated than the precursor in young fruit as compared to bark tissue [67]. Thus, these miRNAs might be processed differently in different tissues.

### 6.2. Pinaceae

Conserved and novel sRNAs have been identified in multiple conifers including *Pinus taeda* (loblolly pine), and *Pinus contorta* (lodgepole pine), *Pinus abies* (Norway spruce), and *Larix leptolepis* (Japanese larch) [69,70,71,72].

#### 6.2.1. *P. taeda*

In *Pinus taeda*, miRNAs are categorized into two classes viz. i) conserved class (*pta-miR156, pta-miR159, pta-miR160* and *pta-miR319*) and ii) novel class which contains miRNAs that are loblolly pine-specific (*pta-miR946*-*pta-miR952*). Length distribution showed the accumulation of 21-nt miRNAs in needles, stems and roots [71]. Genes for targeted function such as SBP-domain protein, peptidyl-tRNA hydrolase-like targeted by pta-miR156, programmed cell death 6 protein-like, MYB targeted by pta-miR159, and disease resistance protein targeted by pta-miR946 were identified and experimentally validated using 5′-RACE with the mRNAs extracted from xylem (Table 1). Like most plant miRNAs, pta-miRNAs also direct cleavage primarily to a site corresponding to the 10th nucleotide position from the 5′ end. However, the cleavage site varies among different pta-miRNAs [69]. For example, *pta-miR159* (target MYB) and *pta-miR946* (target disease resistance protein) cleave at the 9th nucleotide; *pta-miR951* (target Non-protein coding genes) cleaves at the 16th nucleotide [71]. There are 12 miRNAs unique to miRBase including *pta-miR946a*, *pta-miR1432*, *pta-miR444d*, *pta-miR1309-pta-miR1316*, *pta-miR1319a*, and *pta-miR1320* (http://www.mirbase.org).

#### 6.2.2. *P. contorta*

HTS-sRNA provides the means to characterize and qualitatively profile highly conserved small regulatory RNAs of *P. contorta*. The length distribution of *P. contorta* showed the abundance of 21-nt sRNAs, followed by 22-nt, 20-nt, while occurrence of 24-nt RNA is small [72]. There are 51 miRNA families specific to *P. contorta*. *P. contorta* possess conserved miRNAs such as *miR950*, *miR946*, and *miR1309-miR1316*.

#### 6.2.3. *L. leptolepis*

Japanese larch is an important tree in China, Japan and Europe. Overall, over 150 miRNAs found to be differentially expressed in embryogenic and non-embryogenic callus. Four abiotic stress-induced miRNAs (*miR159, miR169, miR171, miR172*) were largely expressed in embryogenic callus, but almost undetectable in the non-embryogenic callus [73]. Numerous differentially expressed mRNAs and miRNAs in *L. leptolepis* are related to reactive oxygen species (ROS) homeostasis and cell cycle regulation [74] (Table 1). Furthermore, the mRNA–miRNA interaction network exploited several thousand potential target genes for over 200 miRNAs [74].

Although limited information is available among sRNAs, hormone signaling, and dormancy regulation in gymnosperm embryos, Zhang et al. [75] investigated the roles of the endogenous “sRNAome” in dormant and germinated embryos in *Larix leptolepis*. HTS-sRNA showed the presence of over 50 conserved miRNAs belong to 38 families, 3 novel miRNAs, and 16 acceptable miRNA candidates, many of which were upregulated in germinated embryos relative to dormant embryos. HTS-sRNAs of *L. leptolepis* revealed a 24-nt length bias in dormant embryos and a 21-nt bias in germinated embryos. The length bias might be associated with distinct levels of RNA-dependent RNA polymerase 2 (RDR2) and/or RDR6, which is regulated by hormones.

#### 6.2.4. *P. abies* (Norway spruce)

From young seedlings of Norway spruce, 199 distinct small RNA were obtained, and 98 were unique to spruce only [76] (Table 1). The 21-nt sRNAs were most prevalent, 22-nt sRNAs were considerably less abundant, and 24-nt sRNAs were rare. The length distribution of *P. abies* sRNAs is similar to the other gymnosperms such as *Terebra plicata*, *Araucaria araucana*, *Pinus strobus*, *Picea glauca*, and *Ginkgo biloba* [80]. *P. abies* constitute several conserved miRNAs such as *pab-miR159*, *pab-miR395*, *pab-miR396a*, *pab-miR*39*6b*, *pab-miR535*, *pab-miR529*, *pab-miR947*, *pab-miR949*, and *pab-miR951* [76]. A large proportion of the total miRNAs were novel, nonconserved miRNAs. Some of the conserved miRNAs in *P. abies* appeared to be established from introns and were expressed during mRNA maturation [81].

The identified miRNAs fall into eleven miRNA families. Most of the identified in *P. taeda* and *P. trichocarpa* are similar to known miRNAs and only a few matched those of other plant species [72,77]. The differential expression of specific miRNAs in *P. abies* suggests their putative participation in epigenetic regulation [76].

### 6.3. Populus Trichocarpa

The sRNAs in *P. trichocarpa* have been studied thoroughly using approaches such as Sanger sequencing [82], 454 pyrosequencing and massively parallel pyrosequencing [83] and Illumina SBS sequencing technology [77]. The frequency distribution of different sRNA size classes varies in different *Populus* clones/varieties. For example, *P. balsamifera* has a clear dominance of 21-nt sRNAs, followed by 22-nt, then 24-nt [84]. In contrast, 24 and 21-nt sRNAs dominate in *P. trichocarpa* [83], in agreement with size distributions in *A. thaliana*, *Zea mays* and *Physcomitrella patens* [85]. Furthermore, these sRNAs are mainly located on chromosome number 19, overlapping a region containing both the proposed sex-determining locus and a major cluster of nucleotide-binding leucine-rich repeat (NBS-LRR) [83].

Networks of sRNA in stem xylem of *P. trichocarpa* were investigated to gain a enhance insight of wood development processes that probably require the coordinated regulation of many genes [82]. Of the 21 miR families discovered, eleven (*ptr-miR156-ptr-miR172*, *ptr-miR319*, *ptr-miR408* and *ptr-miR472*) are also found in *Arabidopsis thaliana* [85]. However, these conserved miRNAs have species-specific developmental expression patterns, suggesting that even the conserved miRNAs may have different regulatory roles in different species [86]. The miRNAs unique to *P. trichocarpa* (*ptr-miR47-ptr-miR482*) have roles in species-specific developmental processes [82]. The differential expression analysis of *P. trichocarpa* miRNAs in woody stems showed that miRNAs are elicit by mechanical stress and may function in among the most critical adaptations for structural and mechanical fitness.

A number of phased siRNA loci have also been identified, a subset of which are predicted to target disease resistance genes (PPR and NBS-LRR) that have been significantly studied in *Populus* [84] (Table 1). Regulatory roles of *P. trichocarpa* sRNAs in response to long-term stress revealed predominant miRNAs that altered in response to salt, dehydration, cold, heat, and mechanical stresses [87]. Recently, HTS of *P. euphratica* leaves found ~200 conserved miRNAs between *P. euphratica* and *P. trichocarpa*, and over 50 new miRNAs belonging to 38 families were identified, representing an increase in the number of known *P. euphratica* miRNAs [78]. Furthermore, miRNA microarray profiles indicated that 104 miRNA sequences were upregulated, and 27 were downregulated during drought stress. More recently, in *P. trichocarpa* xyloglucan endo-transglycosylase/hydrolase enzyme (XTH16) which is crucial for secondary wood formation, cellulose synthase CSLD4 and vascular-related transcription factors such as *VND7* were predicted as a target for xylem-enriched miRNAs such as ptc-miRX50, ptc-miRX41, and ptc-miRX73 [77].

### 6.4. Malus Domestica (Apple)

HTS identified 23 conserved, 10 less-conserved and 42 apple-specific miRNAs or families with distinct expression patterns in differential tissues (Table 1) [79]. Most of the miRNA target genes represent a wide range of enzymatic and regulatory activities. Three miRNAs including *miR159*, *miR828* and *miR858* collectively target surprisingly several MYB genes (~81 in total) potentially involved in diverse aspects of plant growth and development. In addition, apple also has two conserved *trans*-acting small interfering RNA (tasiRNA) gene families with identical yet unique in their target recognition and biogenesis profiles. The tasiRNAs exploits both miRNA and siRNA biogenesis pathways and requires DCL1, DCL6, RDR6 necessary for miRNA and siRNA production [88].

Plant miRNAs have been detected in wide range of tissues in plants including model and crop species and they are differentially expressed among different tissues [61,73,75,76]. Other miRNAs were identified in the phloem sap [63]. Some apple miRNAs are abundant in the phloem tissue such as *miR156*, *miR159*, *miR160*, *miR162*, *miR167*, *miR169*, *miR396* and *miR398* [89]. Phloem associated miRNAs have demonstrated several important roles such as cell-autonomous expression and effects, long-distance signalling in the regulation of the plant nutrient status, function in establishing gradients of gene expression necessary for developmental patterning and stress responses [74,79,82,89]. Apple phloem related miRNA (few of them) were detected in the phloem sap sample from the stylets of woolly apple aphids [89]. Identification and characterization of miRNAs in apple are thus important to completely understand the regulation of transcription factors and other key regulatory genes.

## 7. Targets of Perennial sRNAs

sRNAs are known to be significant in the regulatory mechanisms of a various of biological processes through the repression of translation and cleavage of targeted mRNAs [76,81]. Transcription factors are amongst the widely studied targets of miRNAs that control diverse physiological processes and genes associated with plant development, metabolism and stress responses [90]. Identification of the target genes associated with the miRNAs in perennial plants has been a tremendous challenge as there is no explicit criterion for determining miRNA targets and to validate their biological efficacy [91].

In the peach, over 50 target genes for known miRNAs were identified (Table 1) [67]. Overall, 15 most conserved miRNA families target more than thirty genes, nine less-conserved miRNA families target 29 target genes. Based on the abundance of the targets transcript, five categories have been formed: (I) conserved miRNAs and their conserved targets, (II) novel targets for conserved miRNAs, (III) targets for other known miRNAs, and (IV) peach-specific miRNAs. Most of the determined targets are belong to the transcription factor gene families, including *ARF*, *NAC*, *SPL*, *MYB*, and *GRF*, while others are relevant to auxin signaling (*TIR/AFB*), sRNA binding (*AGO*), sulfate transport (*AST*), redox reactions (*LAC* and *ARPN*), disease resistance (NBS-LRR), RNA editing, splicing and translation (pentatricopeptide repeat-containing proteins (PPR), protein kinase, FAR1-related, plant defense and growth [67].

With the use of bioinformatics approaches, 53 targets are predicted for 11 miRNA families in *P. taeda* [72], including *pta-miR156* and targets SBP-domain protein and peptidyl-tRNA hydrolase-like; *pta-miR159*, targets MYB and programmed cell death 6 protein-like; *pta-miR160*, ARF10 and Aux/IAA protein; *pta-miR319*, acyl-ACP thioesterase; *pta-miR946*, disease resistance protein; *pta-miR947*, pepsin, microtubule-bundling polypeptide and noncoding genes; *pta-miR948*, serine/threonine kinase and pepsin A; *pta-miR950*, noncoding genes and AMP-binding protein; *pta-miR951*, noncoding genes; and *pta-miR952*, multidrug resistance-associated protein and thaumatin-like protein (Table 1). Members of the SPB, MYB and ARF protein families have earlier been determined as targets of miR156, miR159 and miR160, respectively [88].

In *L. leptolepis*, conserved *miR159a* (target MYB33) showed (~5-fold) higher expression in germinated embryos (GE) than in dormant embryos (DE). In *Arabidopsis,* miR159 was triggered by ABA, which regulates transcript levels of *MYB33* and *MYB101* during seed germination [92]. Moreover, the abundance of *MYB33* in *L. leptolepis* found to be higher in GE relative to DE. The expression level of conserved *miR160* (target: *ARF*), *miR166a* (target: *HD-ZIPIII*), *miR397* (target: laccase genes), *miR398* (target: plastocyanin gene) is over 2.0-fold higher in GE than in DE. Adverse regulation of *Auxin response factor 10* (*ARF10*) by *miR160* plays major role in seed germination and post-germination processes [74]. Numerous studies suggested that *miR166* regulates *HD-ZIPIII* expression, mediating indeterminacy in apical and vascular meristems and could be associated in embryo germination [93]. The expression pattern of miR397-laccases coincides with cotyledon extension along with cell propagation [71,94]. Thus, ll-miR397 play vital role in regulating the cell wall thickness during the transition from DE into GE by cleaving the laccase mRNA.

In Norway spruce, the conserved miRNA *pab-miR159a* possibly governing the expression of *PaGaMYB*. In plant, *GaMYB* respond to gibberellin acid (GA) signals and is thus involved in GA transduction pathways, engaged in several growth processes, for instance seed germination and flower development [92,95,96]. Spruce gene, *PaSPB13* found to be regulated by *pab-miRNA* and thus critical in the floral transition and shoot development [97]. Another miRNA, *pab-miR100* regulates the expression of *PaSPT4*, a transcription elongation factor, which impact elongation by RNA polymerase II and affects growth and rRNA synthesis [98].

In *P. trichocarpa*, miRNAs associated with xylem is found to target genes that are known to be important in secondary growth, including vascular-related transcription factors and the critical reaction-wood enzyme xyloglucan endo-transglycosylase/hydrolase [77]. The MTX-specific miRNA, *ptc-miRX50*, is predicted to target *XTH16*, which encodes a xyloglucan endotransglycosylase/hydrolase (XTH), and the NAC domain transcription factor. The NAC assists to the control wood formation in *P. trichocarpa* and directly regulates the expression of a broad range of genes for the formation xylem vessel [99].

## 8. Conclusions

sRNAs are important regulators of developmental transitions in perennial species over annual species. The progression of perennial species by conventional plant breeding approaches has several limitations mainly caused by their considerable degree of heterozygosity, auto-incompatibility and the length of their juvenile phase. Therefore, RNAi-based approaches that use short stretches of viral sequences have emerged as a powerful technology against viral disease in these species. Emerging genome-editing technologies namely characteristic clustered regularly interspaced short palindromic repeats/CRISPR-associated 9 (CRISPR/Cas9) protein, transcription activator-like effector nucleases (TALENs) and zinc finger nucleases (ZFNs) could also be used to improve perennial species resistance to viruses.

RNAi-mediated silencing, based on sequence homology, is an important drawback in developing virus-resistant plants because the scope of virus resistance is restricted to viral strains or group of viruses that are closely associated with high degree of sequence identity (>90%) across the target region. Therefore, a separate construct for individual virus or a single construct of a sequence conserved between various viruses must be designed. Given that miRNAs and siRNAs are important elements of gene regulatory networks, a comprehensive knowledge of the actions of sRNAs relies on the detection of the target genes. Therefore, it would be intriguing to identify the entire set of miRNAs, siRNAs and their targets in perennial plant species using technology such as NGS. Furthermore, experimental validation of predicted targets could be exploited to decipher their importance in developmental and other physiological processes.

Due to the wealth of information provided and the decreased cost, HTS progressively turn out to be one of the most common tools for studying small RNAs [100,101]. HTS platform allows the resolution of closely related sequences and sequence length variations, and analysis of variants without the need for foreknowledge of the sequence and enable identification of new miRNA sequences and permit the profiling of exogenous RNAs in the sample. Questions about the accuracy of HTS for analyzing and identifying sRNAs populations in perennial plants have arisen, mainly due to (I) multiple sequence alignment in the database to similar sequences caused by short read lengths, (II) mapping of miRNA that are associated with miRNA sequence variation (isomiR) and RNA editing, (III) low consistency of microRNA (miRNA) measurement across platforms, and (IV) the origin of those unmapped reads after screening against all endogenous reference sequence databases. Therefore, accurate analysis of HTS-sRNA data remains challenging and requires a comprehensive and customizable pipeline.

## Figures and Tables

**Table 1:** Number of conserved and novel miRNAs in various organisms and their target genes respectively.

Genus	Organisms	Conserved	Novel	Target	Reference
Pinus	*P. taeda*	4	7	SBP-domain protein, peptidyl-tRNA hydrolase-like, MYB, programmed cell death 6 protein-like, ARF10, Aux/IAA protein, acyl-ACP thioesterase, disease resistance protein, pepsin, microtubule-bundling polypeptide, noncoding genes, serine/threonine kinase, AMP-binding protein, multidrug resistance-associated protein and thaumatin-like protein	[69,71]
	*L. leptolepis*	88	16	Scarecrow-like (SCL) transcription factor, apetala2, MYB, NF-YA transcription factor, Basic blue protein, WUS-related homeobox 8, AP2-like ethylene responsive transcription factor BBM2, probable cellulose synthase A catalytic subunit 6, growth-regulating factor 6, superoxide dismutase, ascorbate peroxidase catalase, monodehydroascorbate reductase, peroxidase peroxiredoxin, thioredoxin, peroxidase, TOM1-like protein 2	[73,74,75]
	*P. abies*	101	98	MYB, TIR/P-loop/LRR disease resistance protein-like protein, CC-NBS-LRR resistance-like protein, BIP2_TOBAC Luminal-binding protein 2 (BiP 2), glucose-regulated protein homolog 2-Hsp70 family, thiF family protein, molybdopterin biosynthesis protein, transcription elongation factor – Spt4/zinc ion binding, GAMyb, putative auxin response factor ARF16, homeodomain-leucine zipper protein, Squamosa promoter-binding SBP-domain like protein 13 (SPB13), lipid transporters 4 (LPT4)	[76]
Populus	*P. trichocarpa*	157	33	Glucan synthase-like 12, ribosomal protein S15A, growth-regulating factor 7, Cystathionine beta-synthase (CBS) family protein, ubiquitin-protein ligase 1, maternal effect embryo arrest 22, RNI-like superfamily protein, RING/U-box superfamily protein, Zinc finger, C3HC4 type (RING finger) family protein, ATPase family associated with various cellular activities (AAA), KNOTTED-like homeobox of Arabidopsis thaliana 7, mechanosensitive channel of small conductance-like 6, LRR and NB-ARC domains-containing disease resistance protein, B-box type zinc finger family protein, Exostosin family protein, somatic embryogenesis receptor-like kinase 2, DYNAMIN-like 1E, Vacuolar import/degradation, Vid27-related protein, sigma factor 4, Ankyrin repeat family protein, serine carboxypeptidase-like 35, BTB/POZ domain-containing protein, multidrug resistance-associated protein 2	[77]
	*P. euphratica*	21	26	Electron carrier activity, DNA binding, Transcription factor, SBP-box, Vesicle transport v-SNARE, NADH-ubiquinone oxidoreductase, Cytochrome c oxidase biogenesis protein, Development/cell death domain	[78]
Malus	*M. domestica*	33	42	MYB, Squamosa promoter-binding-like protein, Transcription factor GAMYB, NAC domain-containing protein, Homeobox-leucine zipper protein, Auxin response factor, Argonaute protein, Scarecrow-like protein, Ethylene-responsive transcription factor RAP, Transcription factor TCP4, Auxin signaling F-box protein, 3’-Phosphoadenosine 5’-phosphosulfate synthase, Growth regulating factor (GRF) Protein kinase, Mate efflux family protein, Oligopeptide transporter 2, Mitogen-activated protein kinase kinase 2, Cysteine protease	[79]
Prunus	*P. persica*	47	47	Zinc finger protein, NBS-LRR class disease resistance protein, Pentatricopeptide (PPR) repeat-containing protein, Protein kinase family protein, Translation initiation factor, Esterase/lipase/thioesterase family protein, Allene-oxide cyclase, FAR1-related sequence 3, RNA binding, Catalase, Vernalization independence, DNA binding, Squamosa promoter-binding-like protein, MYB, Auxin response factor, Homeobox-leucine zipper protein, NAC domain-containing protein, Growth-regulating factor, Laccase, Copper ion binding protein, Selenium-binding protein, Cyclin D3, 3-ketoacyl-CoA thiolase, Disease resistance-responsive protein, Vacuolar processing enzyme	[67]
